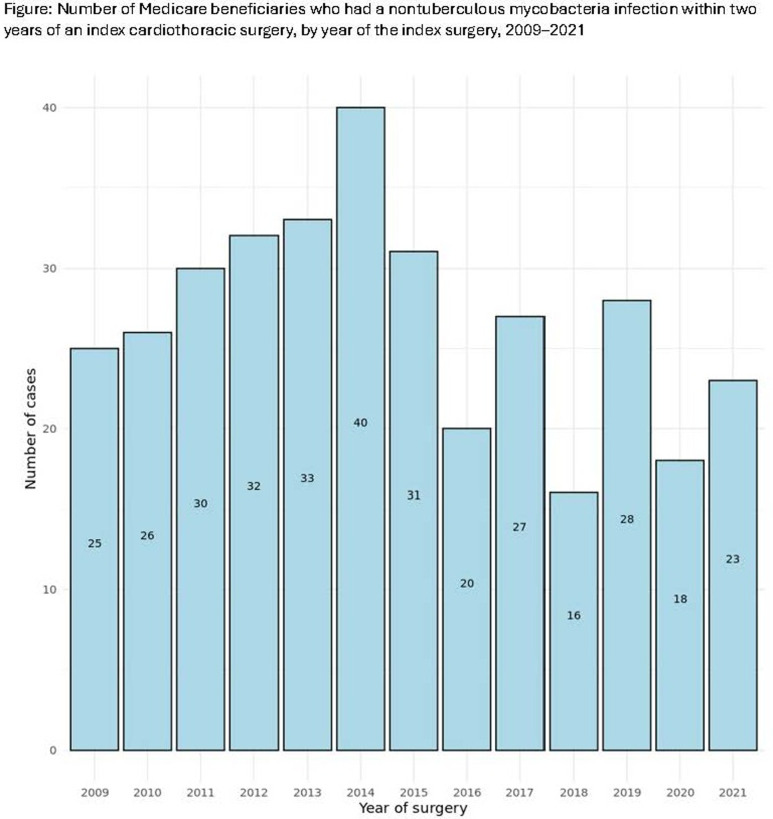# 354 Evaluating meningitis/encephalitis PCR panel performance

**DOI:** 10.1017/ash.2026.10692

**Published:** 2026-06-23

**Authors:** Emily Ussery, Austin Penna, Kelly Hatfield, Joseph Lutgring, Ryan Fagan, Sujan Reddy, Kiran Perkins

**Affiliations:** 1 Centers for Disease Control and Prevention; 2 CDC

## Abstract

**Background:** Nontuberculous mycobacteria (NTM) have caused serious infections following cardiothoracic surgery. In 2015, the first reports of NTM infections attributed to heater-cooler devices used during cardiopulmonary bypass (CPB) led to a prolonged international response. Surveillance and clinical management of these infections is challenging due to the long period (approximately 13–26 months) between exposure and disease presentation. Our objective was to describe the frequency and clinical outcomes of NTM infections following surgery with CPB among U.S. Medicare beneficiaries. **Methods:** We used Centers for Medicare & Medicaid Services claims data for Medicare fee-for-service beneficiaries. The study included adults who had (a) an inpatient claim with a procedure code for surgery requiring CPB between 2009–2021, and (b) an inpatient claim with a non-pulmonary NTM diagnosis code within two years after the index surgery. For beneficiaries with multiple surgeries in 2009–2021, only the first was included. Beneficiaries with an NTM diagnosis code on a claim in the year before the surgery were excluded. Demographic and clinical characteristics among cases and clinical outcomes in the two years after the index surgery were described. **Results:** Overall, 349 NTM case-patients with an index cardiothoracic surgery between 2009-2021 were identified. Cases had a median age of 70 years (interquartile range [IQR]: 65–77) (Table). The number of cases peaked in 2014 (n=40) and was lowest in 2018 (n=16) (Figure). The largest proportion of index surgeries were coronary artery bypass graft surgeries (45.0%), followed by cardiac valve surgeries (33.8%) and ventricular assist device surgeries (19.5%). The median time from index surgery to the first hospitalization with an NTM diagnosis code was 195 days (IQR: 75–416), and 26.9% of cases (n=94) had a diagnosis code for a serious infection (sepsis, endocarditis, or other bloodstream infection) at the time of the NTM diagnosis. During the two years after the index surgery, cases had a median of 4 inpatient hospitalizations and the median cumulative length of inpatient stays was 41 days (IQR: 18–77); 129 cases (37.0%) died, including 51 deaths among cases with a serious infection (54.3%). **Conclusions:** NTM infections following cardiothoracic surgery involving CPB continue to be identified despite heater-cooler device-related interventions. Although this claims-based analysis was unable to assess detailed clinical manifestations or cause of infection, the results suggest a need for continued attention to NTM prevention strategies in patients requiring CPB surgery to prevent these severe infections that are associated with multiple subsequent hospitalizations and high mortality.

**Figure:**
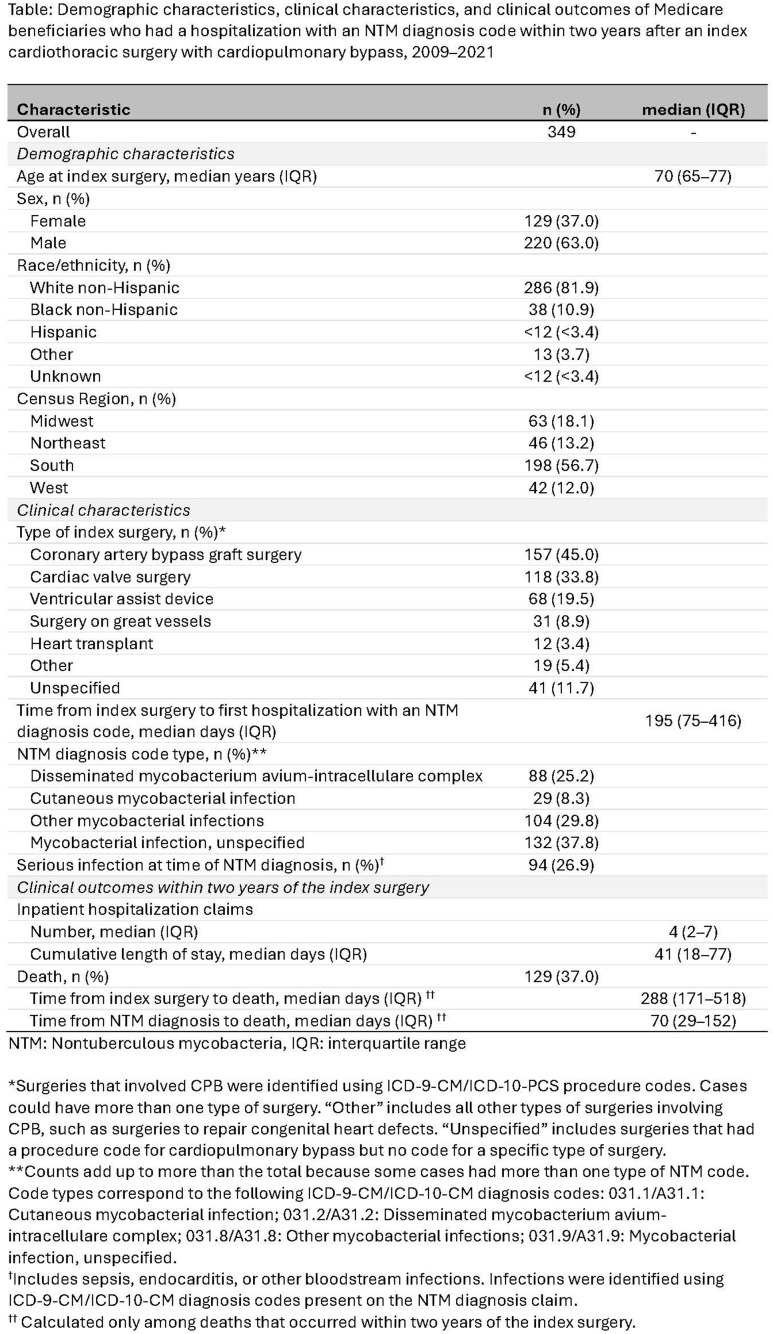

**Figure:**